# Revisiting Azithromycin for Intermittent Preventive Treatment in Pregnancy

**DOI:** 10.4269/ajtmh.24-0734

**Published:** 2025-02-04

**Authors:** Michelle E. Roh, Holger W. Unger, Catherine E. Oldenburg

**Affiliations:** ^1^ Institute for Global Health Sciences, University of California, San Francisco, California;; ^2^ Menzies School of Health Research, Charles Darwin University, Darwin, Northern Territory, Australia;; ^3^ Department of Infectious Diseases, Doherty Institute, Melbourne, Australia;; ^4^ Department of Clinical Sciences, Liverpool School of Tropical Medicine, Liverpool, United Kingdom;; ^5^ Francis I Proctor Foundation, University of California, San Francisco, California;; ^6^ Department of Epidemiology and Biostatistics, University of California, San Francisco, California

In malaria-endemic regions of Africa, the World Health Organization recommends pregnant women receive intermittent preventive treatment in pregnancy (IPTp), which entails providing full treatment courses of a long-acting antimalarial from the second trimester to delivery, with doses given at least one month apart.^1^ Sulfadoxine-pyrimethamine (SP) is the only antimalarial currently recommended for IPTp, but its antimalarial efficacy is threatened by the emergence and spread of SP resistance. Azithromycin (AZ) has been considered as a potential partner drug for IPTp, with the aim of strengthening antimalarial protection while also impacting on other factors associated with adverse birth outcomes. Several clinical trials of AZ-containing IPTp regimens have been conducted, including combinations of AZ with SP,^2^^-^^5^ chloroquine,^6^ dihydroartemisinin-piperaquine (DP),^7^ piperaquine,^8^ and trimethroprim-sulfamethoxazole.^9^

In this issue of the *American Journal of Tropical Medicine and Hygiene*, Luntamo and colleagues report on impacts of adding AZ to IPTp-SP on gestational weight gain (GWG), maternal anthropometrics, and anemia through secondary analysis of trial data.^10^ The trial was conducted in Malawi, an area of high SP resistance,^11^ nearly 20 years ago, when national IPTp policy was to provide 2 doses of SP during pregnancy. In the trial, pregnant women were randomized to 2-dose SP (control), monthly SP, or monthly SP + 2-doses of AZ. In HIV-negative women, monthly SP+AZ increased mean GWG by 23 [95% CI: 5-41] grams/week compared to monthly SP, but there was no difference in GWG between the monthly SP and control arms (-6 [95% CI: -24–12] grams/week). By contrast, among HIV-positive pregnant women, whose mean GWG was 37% lower than that of their HIV-negative counterparts (161 grams/week versus 257 grams/week), monthly SP increased average GWG by 78 [95% CI: 29–126] grams/week compared to control, with an additional 42 [95% CI: -6-91] grams/week increase for those receiving monthly SP+AZ. These findings complement previous analyses of data from the same study,^2^^,^^12^^,^^13^ which showed that monthly SP+AZ reduced risks of preterm delivery by 34% [95% CI: 9%–52%], very preterm delivery by 52% [95% CI: 19%–74%], and low birthweight (LBW) by 39% [95% CI: 7%–60%] compared to 2-dose SP.^2^ Smaller, non-statistically significant reductions in these outcomes were observed when monthly SP was compared to control. Comparison of malaria outcomes showed that increasing the frequency of SP dosing considerably reduced malaria risk, with limited additional benefit from adding AZ: compared to 2-dose SP, the prevalence of PCR-confirmed parasitemia at delivery was lower by 67% [95% CI: 36%–83%] in the monthly SP arm and 77% [95% CI: 52%–89%] in the monthly SP+AZ arm.^13^ However, the risk of anemia did not significantly differ between the three study arms.^10^

Luntamo and colleagues’ collective findings offer valuable insights into the potential impacts of IPTp with SP and AZ on GWG and birth outcomes. As the authors note, these data were from two decades ago, when the prevalence of drug resistance and other factors impacting on outcomes may have differed from those now present. The relevant question in today’s context is whether adding AZ to monthly IPTp-SP provides a benefit. In the study, adding AZ to monthly SP was associated with a 23 gram/week increase in GWG for HIV-negative women. However, similar comparisons were not conducted for malaria, birth, and neonatal outcomes,^2^^,^^12^^,^^13^ as the study was not powered to compare between the monthly SP and SP+AZ arms. Without clear evidence on how this modest increase in GWG translated into clinically meaningful differences in birth outcomes, the potential benefit of adding AZ to IPTp-SP remains uncertain. Further, while the greatest gains were seen in HIV-positive women, these results may not be applicable to the modern HIV-positive pregnant population, with widespread use of antiretroviral therapy^14^ and trimethoprim-sulfamethoxazole (which obviates the use of SP).^15^

Several other trials have investigated the value of adding AZ to monthly IPTp regimens, yielding less favorable results.^5^^,^^7^ A trial from Kenya, Malawi, and Tanzania^7^ assessed the effect of adding a single course of AZ to monthly IPTp with DP among HIV-negative pregnant women, and found no improvements in GWG, preterm delivery, or LBW risk with AZ. Moreover, while both the DP and DP+AZ arms were highly effective in preventing malaria, neither outperformed monthly SP in improving GWG or birth outcomes. Most recently, in a trial from Burkina Faso,^5^ the addition of 2-dose AZ to monthly SP did not result in significant differences in birth outcomes. Considering HIV-positive pregnant women, a trial from Cameroon^9^ evaluated the effect of adding monthly AZ to daily trimethoprim-sulfamethoxazole and found no significant differences in malaria, STI, or birth outcomes. The heterogeneity in observed effects of AZ across studies may be due to a myriad of reasons, including differences in study design, outcome ascertainment, dosing regimens, partner drugs, and trial populations. As a broad-spectrum antibiotic with reported activity against malaria parasites,^16^ sexually transmitted and reproductive tract pathogens,^17^ respiratory tract bacteria,^18^ and also anti-inflammatory properties,^19^ AZ is expected to offer a range of benefits. However, the risks of these infections are likely to vary widely across populations, which may also partially explain the differences observed between study results. Understanding the mechanisms by which AZ improves birth outcomes may provide useful information on for whom AZ-containing IPTp regimens may be beneficial.

Overall, the new analysis suggested that monthly IPTp with SP+AZ increases GWG compared to monthly IPTp with SP, which likely contributes to its positive impact on birth outcomes. However, considering all available studies, it appears that adding AZ to monthly IPTp with SP may be context-dependent. Thus, further research is needed to identify the settings where AZ could improve maternal and infant health, particularly its impacts on maternal infections and preterm delivery. In addition, the benefits of AZ should be weighed against its potential harms (e.g., selection of antimicrobial resistance). Large-scale community trials have shown short-term increases in the prevalence of macrolide-resistant *Escherichia coli*,* Staphylococus aureus*, and* Streptococcus pneumoniae* following mass distribution of azithromycin,^20^^-^^22^ with scarce data available on longer-term impacts following its use and with individual dosing strategies like IPTp.^23^ Currently, evaluations of alternative IPTp combinations are underway, including DP+SP (NCT04336189, NCT05426434), metronidazole with and without DP or SP (NCT04189744), and a large-scale 2×2 factorial trial evaluating antenatal, intrapartum, and infant administration of AZ (NCT03909737).^24^ In the meantime, the available data suggest that monthly IPTp with SP may be effective for improving GWG and birth outcomes across numerous settings.
